# *Psoralea forbesiae* (Psoraleeae, Fabaceae), a new species from the Swartberg Mountains of South Africa

**DOI:** 10.3897/phytokeys.99.24765

**Published:** 2018-05-30

**Authors:** Charles H. Stirton, Abubakar Bello, A. Muthama Muasya

**Affiliations:** 1 Bolus Herbarium, Biological Sciences Department, University of Cape Town, Private Bag X3, Rondebosch 7700, South Africa; 2 Center for Biodiversity and Conservation, Department of Biology, Faculty of Natural and Applied Sciences, P.M.B. 2218, Umaru Musa Yarádua University, Katsina, Katsina State, Nigeria

**Keywords:** Leguminosae, New species, Endemic, *Psoralea*, Psoraleeae, South Africa, Taxonomy

## Abstract

*Psoralea forbesiae* C.H.Stirt., A.Bello & Muasya is a new species of Psoraleeae, Fabaceae. *Psoralea forbesiae* is endemic to the Swartberg Mountains and is a tall densely branched re-sprouting shrub up to 2.5 m, with bluish-green stems and with most parts covered in small crater-like glands, leaves pinnately 3-foliolate, linear-oblong, pale bluish-green, semi-conduplicate, somewhat succulent, glabrous, crowded at the end of bare branches on older stems or distributed along short branches on young shoots, petiolate. A description of *P. forbesiae*, together with photographs and a distribution map are presented.

## Introduction

The predominantly southern African genus *Psoralea* L. is a young lineage (ca. 4 million years old, ± 75 species) which has diversified rapidly within the Fynbos biome and related habitats (Dludlu et al. 2013, Bello 2016, Bello et al. 2017). During an ongoing taxonomic revision of the genus, it became clear that there were a few localities in the Western Cape Province that showed an abnormal range of local variation, which, on further investigation, indicated that hybridisation had occurred and that this represented both F1 and introgressant hybrids. In one study area in the Swartberg Mountains in South Africa (Bello et al. 2018), it was confirmed that introgressive hybridisation had occurred between *Psoralea sordida* C.H. Stirt. & Muasya and an undescribed species which was noted previously in Stirton and Schutte (2012) as a potential new species and is here formally described.

## Materials and methods

All the data and observations were recorded from specimens collected from their natural populations as well as specimens loaned from various herbaria: BOL, NBG and PRE (acronyms following Thiers 2018). Voucher materials from this study were prepared and are deposited in the Bolus herbarium (BOL), University of Cape Town. Morphological measurements of the major diagnostic vegetative and floral characteristics of all the parents and putative hybrids were made immediately after the samples were collected from the field. A list of all the characters measured is given in Bello et al. (2018). Floral parts from herbarium specimens were soaked in water for five minutes and then carefully dissected using a Leica MS5: WILD 308700 stereomicroscope. Descriptions of vegetation types are based on Mucina and Rutherford (2006) and an attempted assessment of the conservation status of the species was based on the IUCN Red List criteria (IUCN 2012).

## Species treatment

### Psoralea forbesiae

Taxon classificationPlantaeFabalesFabaceae

C.H.Stirt., A.Bello & Muasya sp. nov.

urn:lsid:ipni.org:names:60476481-2

Psoralea sp. 15, Stirton & Schutte in Manning & Goldblatt, Strelitzia 29: 574 (2012).

#### Diagnosis.

Similar to *P. axillaris* L., but differs in being a resprouter with numerous shoots emerging from a woody rootstock; older plants producing a cluster of shoots (burst-branching) at the ends of the previous seasons’ terminal shoots giving an untidy habit (versus a much-branched reseeder with single stem, never with burst branching); stems coarsely fissured and greyish with age (versus furrowed, heavily lenticelled and brownish); leaves 3-foliolate; leaflets partially conduplicate, linear-oblong, with raised crater-like glands and scarcely visible veins (versus leaves 3–5-foliolate; flat, lanceolate, distinctly veined with small sunken glands); lateral leaflets symmetrical, 2–3 mm broad (versus lateral leaflets asymmetrical, 3–8 mm. broad); flowers well exerted from leaves, mauve to pale lavender, wings white (versus mostly hidden within leaves, mauve to purple with purple veins, wings mauve); standard white to pale mauve and with a single purple vertical flash plus a few shorter darker veins towards base of standard, apex greenish on front and back (versus mauve with strongly purple veins and violet basal patch, apex not greenish on front and back); wing petals flared outwards (versus wing petals held vertically).

#### Type.

SOUTH AFRICA, Western Cape Province, 12.5 km from Swartberg Pass – Prince Albert Road to Gamkaskloof, 33°21'11.9"S, 21°56'32.3"E, 1417 m, 24 November 2011, flowering, *Stirton & Muasya 13279* (Holotype: BOL!).

#### Description.

**Figure 1.**
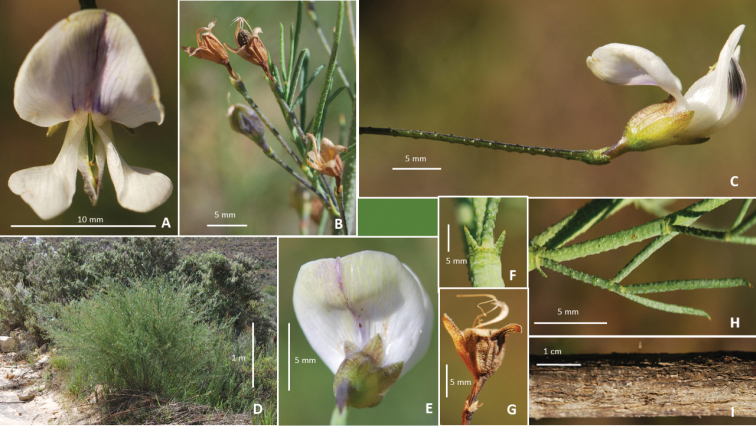
*Psoralea forbesiae* C.H.Stirt., A.Bello & Muasya: **A** front view of flower **B** Fruiting calyces **C** Side view of flower **D** Habit **E** Back of standard **F** Stipule **G** Fruiting calyx **H** Leaf **I** Stem. Photographs by Charles Stirton and Abubakar Bello. Voucher *Stirton & Muasya 13279* (BOL).

Tall densely branched shrubs to 2.5 m, resprouter, bluish-green, most parts covered in small crater-like glands; mature plants can be hemispherical. *Stems* many, green turning bluish- green to brown with age, coarsely fissured, older plants produce burst branching at the ends of previous season's seasonal shoots giving an untidy habit; seasonal shoots glaucous, glabrous, densely covered in small raised crateriform glands. *Leaves* pinnately 3-foliolate, yellowish-green, semi-conduplicate, semi-succulent, glabrous, crowded at the end of bare branches on older stems or distributed along short branches on young shoots, petiolate. *Stipules* triangular, short, straight, stiff, erect, fused near their base, glabrous, glandular, rapidly senescent, persistent, shorter than petiole. *Leaflets* linear-oblong, symmetrical, glabrous, bluish-green; apex acute, tip deflexed, terminal leaflets 20–30 × 1.4–3.0 mm, laterals (12) 15–24 × 1.5–3.0 mm, petioles (7) 10–11 mm long; rachis 2–5 mm long, small, terminal leaflet longest. *Inflorescences* axillary, borne in upper axils of seasonal shoots, 1 (2) flowers per axil, pedunculate, pedicel 4–5 mm long, shorter than calyx tube; peduncles rigid, 26–35 mm long, longer than the subtending leaf; cupulum terminal, 3-fid, teeth equal, triangular, minute, warty, glabrous, 1.7–1.8 mm long. *Flowers* 8-11 mm long, white to pale mauve, held above the foliage. *Calyx* 5-8 mm long; ribbed, densely glandular, glands smaller on triangular teeth; lobes equally developed, shorter than the calyx tube, glabrous, carinal lobe slightly wider; ribs and tube sometimes flushed purple. *Standard* 7–8 × 10–11 mm, white or pale mauve fading towards margins, with a purple vertical flash tapering to the apex and some basal veins purplish, apex greenish on front and back. *Wing petals* 8–11 × 4–5 mm, white, tips sometimes pale lavender, longer than keel, blade flared outwards, sculpturing present. *Keel* 7–8 × 4 mm, white but apically suffused with dark violet-purple on inner apex. Pistil stipitate, ovary glabrous but sparsely covered in club-shaped glands, style glabrous, curved upwards, thickened at point of flexure. *Fruits* 1-seeded, papery, enclosed within calyx, surface reticulate. *Seeds* black (Fig. 1).

#### Distribution, habitat and ecology.

**Figure 2.**
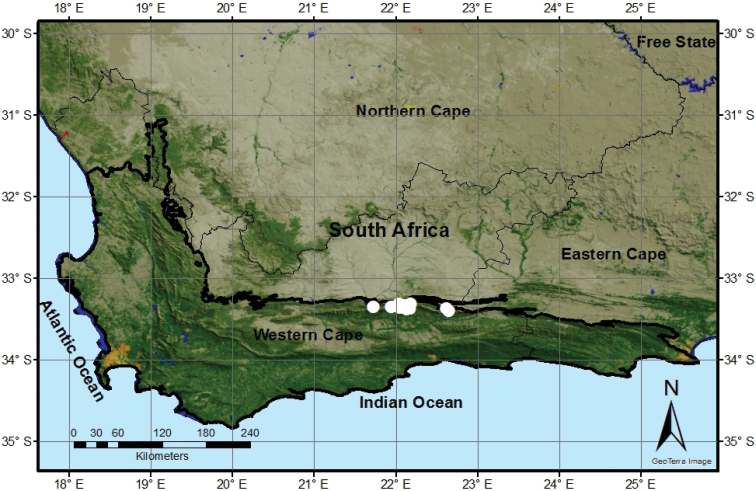
The geographical distribution of *Psoralea forbesiae* (white circles).

*Psoralea forbesiae* is a locally common species known only from the mid- to upper altitudes on the southern slopes and plateau of the Swartberg Mountains of the Western Cape Province (Fig. 2). It occurs in seepages, gulleys and along streams in mountain fynbos between 1200–1700 m (a.s.l.). It is restricted to the South Swartberg Sandstone Fynbos and North Swartberg Sandstone Fynbos vegetation types (FFs 23 & FFs 24) (Mucina and Rutherford 2006). It forms part of an introgressive hybrid swarm with *P. sordida* on the flanks of the road leading up the southern slopes of the Swartberg Pass (Bello et al. 2018). The flowers are visited by black Megachilid and Xylocopid bees.

#### Phenology.

Flowering takes place between November and March.

#### Etymology.

The specific epithet *forbesiae* honours Scottish born Helena Madelain Lamond Forbes (1900–1959) who immigrated to South Africa with her parents when young. She worked at the National Herbarium in Pretoria, visited Kew Gardens for one year and ended up as the Curator of the Natal Herbarium (NH). She wrote local floras of Isipingo and Malvern districts in Natal but is best known for her revisions of *Tephrosia* and *Psoralea* in South Africa (see Gunn and Codd 1981, Glen and Germishuizen 2010).

#### Preliminary conservation status.

More information is needed to evaluate the conservation status of this species as it is part of an introgressive hybrid swarm with *P. sordida* (Bello et al. 2018). Based on the IUCN Red List Categories and Criteria guidelines the new species is treated as “Data Deficient (DD)” (IUCN 2012).

#### Related species.

*Psoralea forbesiae* is part of the *Psoralea verrucosa* complex with special affinities to *P. triflora* Thunb. and *P. verrucosa* Willd. It has been confused in the past with *P. verrucosa* and usually named as that species. However, *P. verrucosa* is an allopatric species from the Cederberg region (versus Swartberg Mountains), with glaucous and prominently warty stems and leaves (versus bluish-green stems and leaves covered in small raised crateriform glands) and multi-flowered pedunculate inflorescences (versus single-flowered axillary inflorescences). *Psoralea triflora* is an allopatric lowland coastal species of shorter stature (<1.5 m) and differs from *P. forbesiae* in its flat, 1.0–1.7 mm broad, keeled leaflets with impressed glands (versus semi-conduplicate 1.4–3.0 mm broad leaflets densely covered in small raised crateriform glands); peduncles 10–15 mm long (versus peduncles 26–35 mm); and with mauve standards with purple veins, prominent central purple flash and nectar guide, back purple (versus standard white to pale mauve with a single purple vertical flash plus a few shorter darker veins towards base of standard, white with apex greenish on front and back). It is difficult to name some material belonging to *P. forbesiae* in the Swartberg Mountains owing to the presence of an introgressive swarm there. *Psoralea sordida*, with which it hybridises, is a lanky 1–2-stemmed shrub with erect short virgate branches in its upper parts (versus many-stemmed, densely branched large shrubs tending to hemispherical in shape); with digitately (3)5(7)-foliolate glabrous green leaves with sunken glands (versus pinnately 3-foliolate bluish green leaves with raised crateriform glands); leaflets linear-lanceolate, 0.2–0.3 mm wide (versus leaflets linear-oblong, 1.5–3.0 mm wide); 3-flowered axillary inflorescences shorter than the subtending leaves with stout and rigid 2–4 mm long peduncles (versus 1(2)-flowered inflorescences longer than the subtending leaves, with filiform and 26–35 mm long peduncles); and calyx lobes equally developed (versus unequally developed).

#### Specimens examined.

Cement bridge across river just west of Bothashoek in Groot Swartberg Mountains, (3321CB), 10 March 2015, *Du Preez 29* (BOL).

Top of Swartberg Pass, Swartberg Mountains (3322AC), 10 December 1978, *Stirton 10308, 10331* (PRE).

Swartberg Pass, Swartberg Mountains (3322AC), 17 February 2014, *Bello, Stirton, Muasya & Chimphango 182, 207, 208, 209, 223, 224, 225, 226, 227* (BOL).

1.6 km from Swartberg Pass – Prince Albert Road to Gamkaskloof, (3322AC), 24 February 2011, *Stirton & Muasya 13272* (BOL).

Bassonsrust, Upper Cango Valley, (3322AC), 29 March 1975, *Moffet 672* (NBG).

8 km from Prince Albert – Oudtshoorn road to Die Hel, (3322AC), 1 January 2008, *Muasya & Stirton 3592* (BOL).

## Supplementary Material

XML Treatment for Psoralea forbesiae
